# Podocyte-specific Rac1 deficiency ameliorates podocyte damage and proteinuria in STZ-induced diabetic nephropathy in mice

**DOI:** 10.1038/s41419-018-0353-z

**Published:** 2018-03-01

**Authors:** Zhimei Lv, Mengsi Hu, Minghua Fan, Xiaobing Li, Jiangong Lin, Junhui Zhen, Ziyang Wang, Haijun Jin, Rong Wang

**Affiliations:** 1 Department of Nephrology, Shandong Provincial Hospital Affiliated to Shandong University, Jinan, China; 2 Department of Obstetrics and Gynecology, The Second Hospital of Shandong University, Jinan, China; 3 Institute of Basic Medicine, Shandong Academy of Medical Sciences, Jinan, China; 4 Department of Pathology, School of Medicine, Shandong University, Jinan, China; 5 Department of Internal Medicine Clinic, Shandong Provincial Hospital Affiliated to Shandong University, Jinan, China

## Abstract

Activation of Ras-related C3 botulinum toxin substrate 1 (Rac1) has been implicated in diverse kidney diseases, yet its in vivo significance in diabetic nephropathy (DN) is largely unknown. In the present study, we demonstrated a podocyte-specific Rac1-deficient mouse strain and showed that specific inhibition of Rac1 was able to attenuate diabetic podocyte injury and proteinuria by the blockade of Rac1/PAK1/p38/β-catenin signaling cascade, which reinstated the integrity of podocyte slit diaphragms (SD), rectified the effacement of foot processes (FPs), and prevented the dedifferentiation of podocytes. In vitro, we showed Rac1/PAK1 physically bound to β-catenin and had a direct phosphorylation modification on its C-terminal Ser675, leading to less ubiquitylated β-catenin, namely more stabilized β-catenin, and its nuclear migration under high-glucose conditions; further, p38 activation might be responsible for β-catenin nuclear accumulation via potentiating myocyte-specific enhancer factor 2C (MEF2c) phosphorylation. These findings provided evidence for a potential renoprotective and therapeutic strategy of cell-specific Rac1 deficiency for DN and other proteinuric diseases.

## Introduction

Diabetic nephropathy (DN) is one of the leading causes for chronic kidney diseases^1^. Podocytes are terminally differentiated epithelial cells of the glomerulus, essential for the maintenance of an intact glomerular filtration barrier (GFB), and damaged podocytes are a key contributor to the onset of proteinuria and the progression of DN^2–4^. To date, mechanisms that govern diabetic podocyte damage and kidney injury remain poorly understood.

Rac1, a member of Rho family small GTPases, is a multi-functional molecule implicated in various cellular processes involving cell adhesions, proliferation, and plasticity^5^. Abnormal Rac1 signaling is involved in ROS production and inflammatory responses and reportedly linked to a number of debilitating human diseases, including cancer, diabetes, and kidney disorders^5–8^. Kolavennu et al. demonstrated in vivo targeting RhoA signaling, another pivotal member of Rho GTPases, could ameliorate albuminuria in a rodent model of diabetes via downstream signaling ROCK^9^. And we previously showed in vitro that Rac1 interference was protective against high-glucose (HG)-induced podocyte damage^10^, hence we postulated that manipulating Rac1 expression might as well be of a curative potentiality in vivo. Notably, systemic KO of Rac1 would lead to embryonic lethality in mice due to germ-layer formation defects^11, 12^. Thus in the present study, we described a podocyte-specific Rac1-deficient mouse strain and generated diabetes models in these mice, aiming to uncover a renoprotective and therapeutic role of cell-specific Rac1 deficiency in DN and related podocyte damage.

Among many factors implicated in the pathogenesis of DN, p38 MAPK (p38), which belongs to MAPK family, is typically involved in diabetes as a critical mediator of inflammatory reactions and mitochondrial malfunction^13^. Hyperphosphorylated p38 is found in renal proximal tubular epithelial cells (PTECs) and contributes to their epithelial–mesenchymal transition (EMT)^13, 14^. Aberrant p38 phosphorylation was also correlated with the modulation of podocyte cytoskeletal dynamics^15, 16^. Additionally, p38 could be activated by PAK1, a major downstream target of Rac1, as reported in several cancer cell lines and tracheal smooth muscle cell^17, 18^. However, interplays between Rac1/PAK1 and p38 in diabetic podocytes and how would these interactions contribute to podocyte damage and proteinuria is not fully clarified.

It was reported that Rac1 activation controlled nuclear localization of β-catenin, a key intracellular signal transducer involved in kidney fibrosis, during canonical Wnt signaling, depending on phosphorylation at its Ser191 and Ser605^19^. In our previous study, Rac1/PAK1 activation was sufficient to trigger enhanced β-catenin dephosphorylation in podocytes, which later provoked elevated Snail expression upon HG stimulation^10^. Intriguingly, p38 could also regulate β-catenin signaling by inactivation of GSK3β in mouse F9 cells^20^, whereas little information is available on crosstalk between p38 and β-catenin in podocytes. Recent studies indicated that C-terminus domain of β-catenin might constitute the minimum region necessary for β-catenin shuttling between the cytosol and nucleus^21^. Thus it would be of interest to further identify whether there are modifications at β-catenin C-terminus in podocytes under HG conditions. Collectively, we tested our hypothesis in the present study that podocyte-specific transgenic ablation of Rac1 might be renoprotective against podocyte damage and proteinuria via prohibiting a signaling cascade of Rac1/PAK1/p38; and that epic activation of this signaling might also contribute to β-catenin activation and nuclear translocation in damaged podocytes both in vivo and in vitro.

## Results

### TG mice characterization

**Fig. 1:**
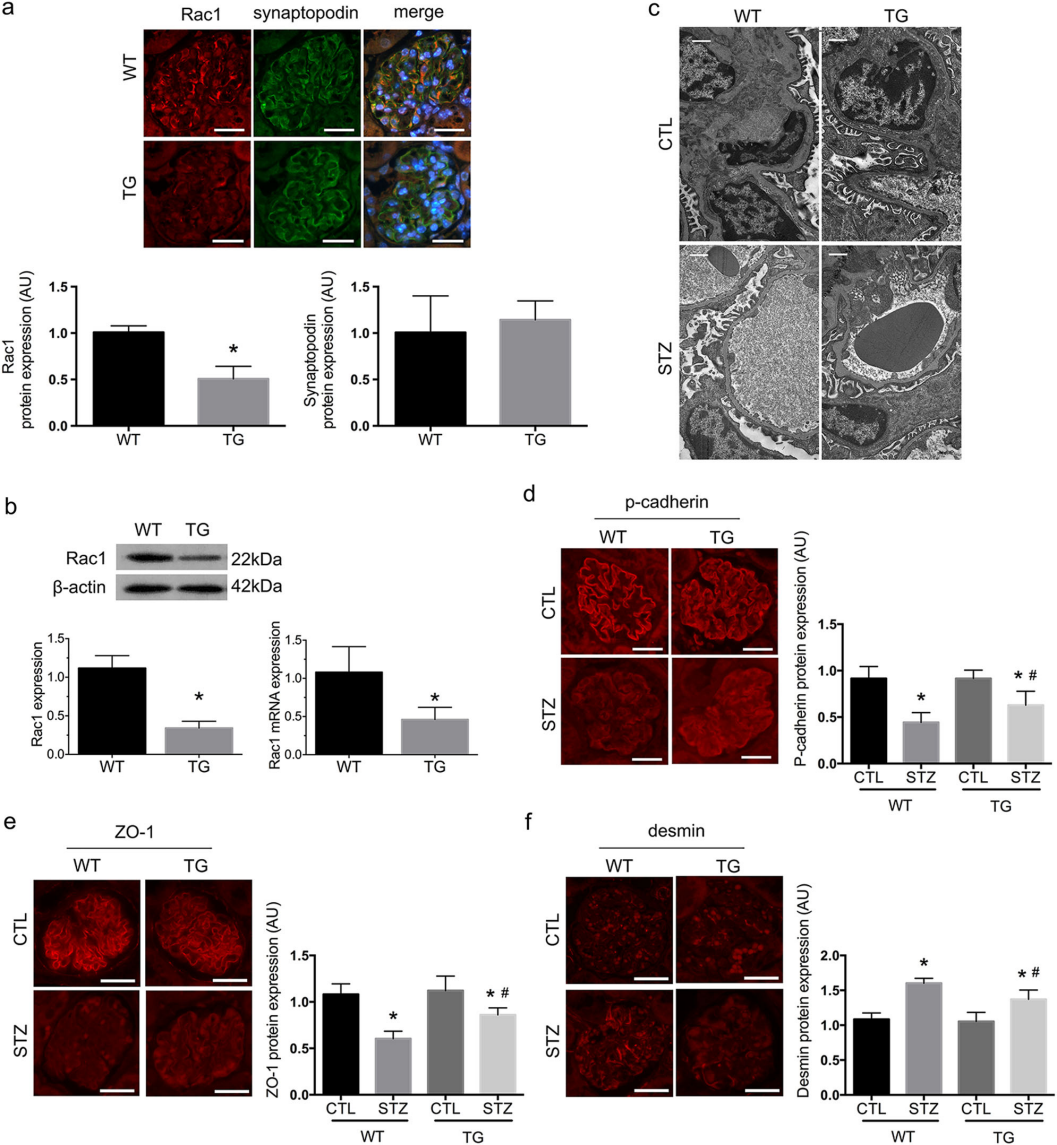
Characterization of podocyte-specific Rac1 TG mice and effects of podocyte Rac1 deficiency on diabetic glomeruli at  6 weeks. **a** Rac1 protein expression was determined by immunofluorescence. Rac1 was reduced in glomeruli of TG mice, in comparison with WT controls. Synaptopodin was double stained to define the edge of glomerular podocytes and no distinguishable differences were observed between the two groups. Values denote the mean ± s.d.; ^*^P < 0.01 vs. WT. Scale bar = 100 μm. **b** Determination of Rac1 levels by western blot and real-time PCR in primary podocytes. Graphs showed lower expression of Rac1 on both protein and mRNA levels in TG mice, compared with WT mice. Values denote the mean ± s.d.; ^*^P < 0.05 vs. WT. **c** Podocyte FP effacement on TEM at 6 weeks. A mild degree of FP retraction was demonstrated in WT-STZ mice, which was attenuated in TG-STZ mice. Scale bar = 1 μm. **d**, **e** Podocyte-specific markers p-cadherin and ZO-1 were detected by immunofluorescence. In CTL mice, p-cadherin and ZO-1 were strongly stained in a linear setting around the glomeruli. There were decreased staining of both p-cadherin and ZO-1 in diabetic WT mice, which were reinstated in diabetic TG littermates. Values denote the mean ± s.d.; ^*^P < 0.01 vs. CTL, ^#^*P* < 0.05 vs. WT-STZ. Scale bar = 100 μm. **f** A mesenchymal marker desmin was simultaneously detected in glomeruli of WT-STZ mice, which was absent in CTL mice; Rac1 deficiency ameliorated the de novo expression of desmin. Values denote the mean ± s.d.; ^*^P < 0.01 vs. CTL, ^#^*P* < 0.01 vs. WT-STZ. Scale bar = 100 μm. AU arbitrary units, TG transgenic, WT wild type, CTL control

TG mice were characterized by immunofluorescence of the kidney cortex, and real-time PCR and western blot analysis of primary cultured podocytes. Immunofluorescence demonstrated a marked decrease in Rac1 staining in TG glomerular podocytes (*P* < 0.01) without altering synaptopodin expression (*P* > 0.05) between wild-type WT and TG mice (Fig. 1a), or podocyte numbers (Figure S) (*P* > 0.05). In primary podocytes, Rac1 expression was significantly reduced in TG mice compared with WT mice on both protein and mRNA levels (Fig. 1b) (*P* < 0.05). There were no distinguishable differences in hair growth or appearance between WT and TG mice (Figure S); and metabolic data showed neither tendency of spontaneous proteinuria or diabetes, nor differences between two mice strains on weight and SBP levels (Table 1) (*P* > 0.05).

**Table 1:** Metabolic data

	Time (week)	*n*	WT-control	WT-STZ	TG-control	TG-STZ
Weight (g)	4	10	20.82 ± 1.201	17.48 ± 2.838^*^	20.72 ± 2.56	20.11 ± 3.048^#^
	6	11	21.682 ± 1.491	17.545 ± 2.66^*^	21.855 ± 0.856	21.364 ± 2.279^#^
	8	9	23.756 ± 1.456	18.256 ± 1.978^*^	23.967 ± 1.133	21.278 ± 2.540^#^
	12	10	30.51 ± 3.975	18.09 ± 3.229^*^	30.32 ± 3.631	20.49 ± 3.445
Blood glucose (mmol/l)	4	10	7.07 ± 1.617	25.1 ± 4.482^*^	7.14 ± 1.977	23.03 ± 3.367
	6	11	7.2 ± 2.106	21.673 ± 3.909^*^	7.109 ± 1.801	20.181 ± 2.342
	8	9	7.211 ± 2.596	28.4 ± 3.094^*^	7.356 ± 1.882	27.022 ± 2.271
	12	10	7.79 ± 2.424	26.85 ± 5.280^*^	7.82 ± 1.615	25.24 ± 5.5572
SBP (mmHg)	4	10	107.1 ± 3.071	107.7 ± 3.653	107.8 ± 7.7143	108.1 ± 6.4885
	6	11	107.91 ± 4.482	107.82 ± 6.432	107.82 ± 5.419	108.45 ± 5.786
	8	9	107.89 ± 7.424	108.56 ± 4.096	107.67 ± 5.788	107.89 ± 8.038
	12	10	108.4 ± 6.883	108.2 ± 6.713	108 ± 1.9441	107.9 ± 4.932
UTP (μg/24 h)	4	10	26.52 ± 2.677	27. 99 ± 3.027	26.65 ± 2.521	27.21 ± 2.961
	6	11	26.255 ± 3.191	28.891 ± 3.051	26.327 ± 2.246	28.409 ± 3.762
	8	9	26.544 ± 2.671	45.956 ± 7.41^*^	26.311 ± 4.378	37.378 ± 7.109^#^
	12	10	26.09 ± 2.798	134.48 ± 20.549^*^	26.78 ± 2.9139	99.55 ± 16.501^#^

Values represent means ± s.d.

*P < 0.01 vs. control mice; ^#^*P *< 0.05 vs. WT-STZ

### Dedifferentiation of glomerular podocytes was found preceding the onset of proteinuria in diabetic mice

**Fig. 2:**
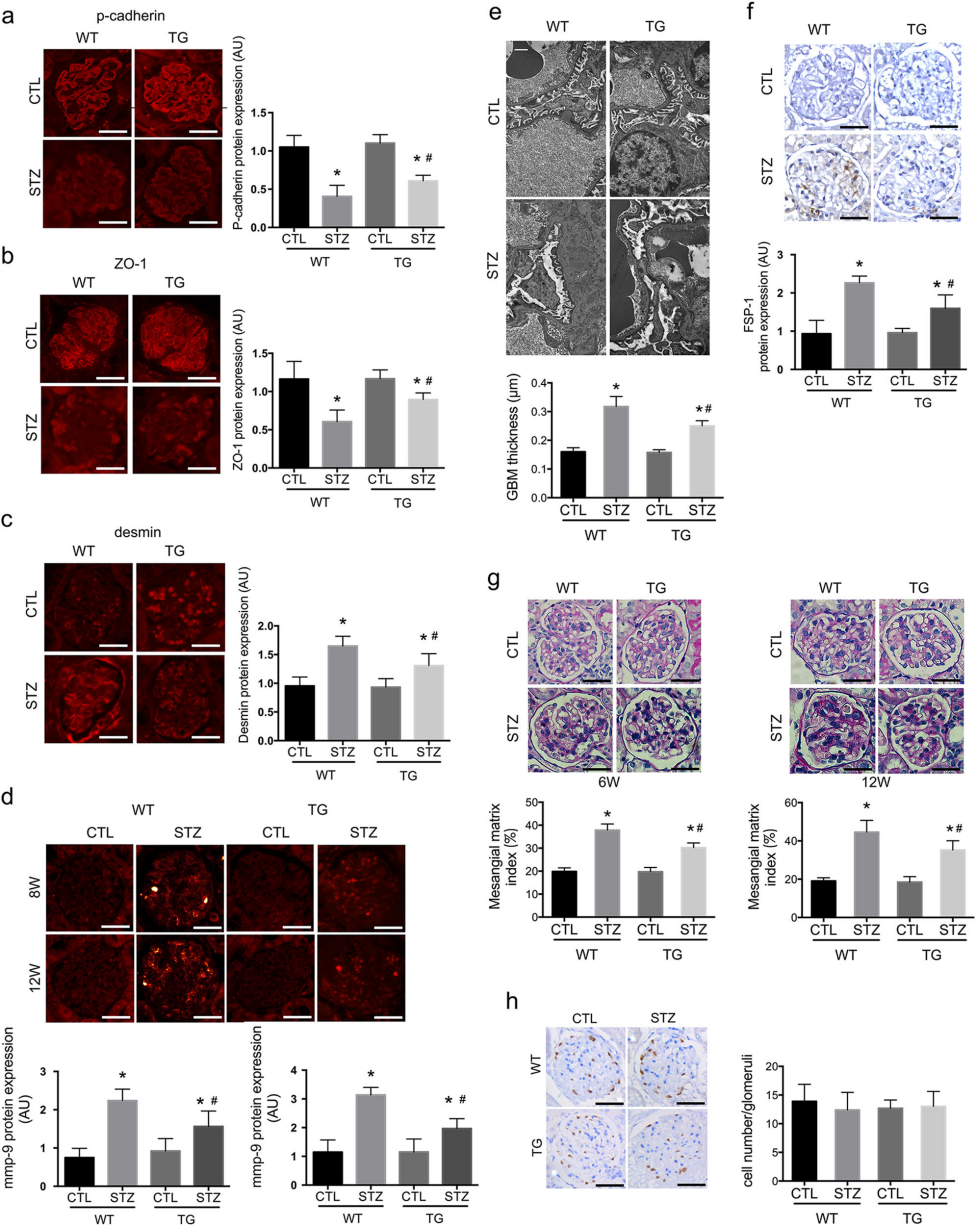
Effects of podocyte Rac1 deficiency on glomeruli at  12 weeks. **a**, **b** At 12 weeks, staining of podocyte-specific markers p-cadherin and ZO-1 markedly decreased in WT mice; Rac1 deletion partially restored the expression of both proteins. Values denote the mean ± s.d.; ^*^P < 0.01 vs. CTL, ^#^*P* < 0.05 vs. WT-STZ. Scale bar = 100 μm. **c** At this time point, desmin expression was strongly stained in glomerular podocytes and parietal epithelial cells in WT diabetic mice, which was prohibited in TG-STZ mice. Values denote the mean ± s.d.; ^*^P < 0.01 vs. CTL, ^#^*P* < 0.01 vs. WT-STZ. Scale bar = 100 μm. **d** At 8 and 12 weeks, mmp-9 expression was positively stained in mesangial cells and podocytes in WT-STZ mice, which was decreased in diabetic TG mice. Values denote the mean ± s.d.; ^*^P < 0.01 vs. CTL, ^#^*P* < 0.01 vs. WT-STZ. Scale bar = 100 μm. **e** Podocyte effacement on TEM. Podocyte FPs were found extensively effaced in WT-STZ mice, but were markedly rescued in TG-STZ counterparts. Scale bar = 1 μm. **f** De novo expression of a fibroblastic hallmark FSP-1. In CTL glomeruli, FSP-1 was negatively stained in glomerular podocytes; 12 weeks of diabetes led to de novo expression of FSP-1, which was partially diminished in TG-STZ glomeruli. Values denote the mean ± s.d.; ^*^P < 0.01 vs. CTL, ^#^*P* < 0.01 vs. WT-STZ. Scale bar = 100 μm. **g** Mesangial and glomerular area by PAS staining. At 6 weeks, PAS staining showed mesangial areas were mildly expanded, with swelled endothelial cells and capillaries stenosis, in WT-STZ mice (left) and at 12 weeks, there occurred moderately mesangial proliferation and matrix accumulation, capillaries stenosis and parietal endothelial proliferation, indicative of advanced renal injury (right); these mutations were attenuated in TG diabetic mice at either time points. Values denote the mean ± s.d.; ^*^P < 0.01 vs. CTL, ^#^*P* < 0.01 vs. WT-STZ. Scale bar = 100 μm. **h** Podocyte numbers were determined by WT staining at 12 weeks. The graph showed no remarkable significance was calculated among four groups. Values denote the mean ± s.d.; *P* > 0.05 vs. CTL. Scale bar = 100 μm. AU arbitrary units, TG transgenic, WT wild type, CTL control

By generating diabetes models, we showed significant differences between WT diabetic mice and their nondiabetic controls in both weight and blood glucose (BG) at 4, 6, 8, and 12 weeks after the onset of diabetes (Table 1) (*P* < 0.05). In WT-STZ mice, TEM demonstrated a mild degree of foot process (FP) effusion at 6 weeks (Fig. 1c), when p-cadherin and ZO-1 began to show decreased staining (*P* < 0.01) (Fig. 1d, e), and a mesenchymal marker desmin presented positive staining (*P* < 0.01) (Fig. 1f). Mutations of these proteins were aggravated as diabetes progressed after 12 weeks (*P* < 0.01) (Fig. 2a–c). And mmp-9 was positively stained in glomerular podocytes and mesangial cells at 8 and 12 weeks (*P* < 0.01) (Fig. 2d). At 12 weeks, FPs were extensively effaced (Fig. 2e); and there was de novo expression of FSP-1 expression observed in podocytes (*P* < 0.01) (Fig. 2f). Moreover, proteinuria occurred at 8 weeks, succeeding theses abnormalities in glomerular podocytes, which was exacerbated at 12 weeks (Table 1) (*P* < 0.05).

### Podocyte-specific knockdown of Rac1 was protective against diabetic podocyte injury and proteinuria

At 6 weeks, PAS staining showed mesangial areas mildly expanded, with swelling endothelial cells and capillaries stenosis, in WT-STZ mice (Fig. 2g) (*P* < 0.01). At 12 weeks, there occurred moderately mesangial proliferation and matrix accumulation, capillaries stenosis and parietal endothelial proliferation, indicative of advanced renal injury (*P* < 0.01) (Fig. 2g). These mutations were attenuated in TG diabetic mice at either time points (*P* < 0.01) (Fig. 2g). Rac1 deficiency partially preserved podocyte markers: staining of p-cadherin (*P* < 0.05) and ZO-1 (*P* < 0.01) were both rectified, with diminished desmin staining compared with WT-STZ mice at 6 (*P* < 0.01) (Figs. 1d, e and 2a, b) and 12 weeks (Figs. 1f and 2c). Mmp-9 staining (*P* < 0.01) (Fig. 2d) as well as FSP-1 (*P* < 0.01) (Fig. 2f) was both abolished in TG-STZ mice, in comparison with their WT diabetes littermates at the indicated time points. By TEM, we found an early alleviation of FP retraction and fusion in Rac1-depleted diabetic glomeruli at 6 weeks compared with WT-STZ group (Fig. 1c). At 12 weeks, process effacement was largely restored with ameliorated GBM in TG-STZ mice (Fig. 2e). In line with these observations, proteinuria was attenuated in TG-STZ mice at 8 and 12 weeks as soon as podocyte proteins were reinstated (Table 1) (*P* < 0.05), without alterations of BG or systolic blood pressure (SBP) (Table 1) (*P* > 0.05). The weight loss was also rescued in TG diabetic mice compared with WT hyperglycemic littermates at 4, 6, and 8 weeks (Table 1) (*P* < 0.05). Moreover, WT-1 staining revealed that podocyte numbers were not altered among four groups at 12 weeks (Fig. 2h) (*P* > 0.05).

### Rac1/PAK1/p38 signaling pathway was activated in vivo and in vitro

**Fig. 3:**
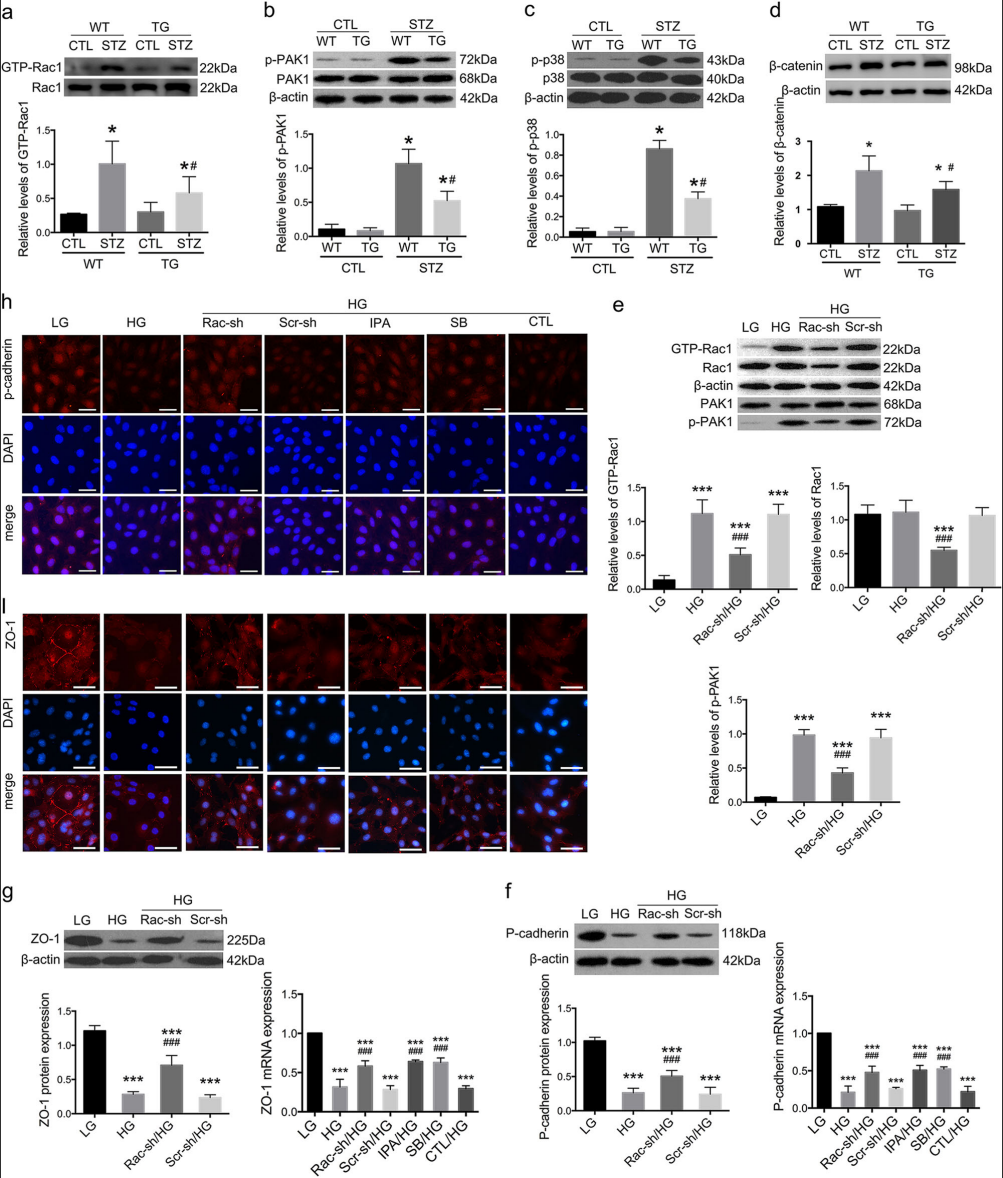
Activation of Rac1/PAK1/p38 signaling cascade in vivo and in vitro. **a**–**d** Activation of Rac1, PAK1, p38, and β-catenin in diabetic mice. GTP-Rac1 was found significantly increased in WT diabetic mice, which was dampened by Rac1 inhibition. Western blot showed significant elevation in PAK1 and p38 phosphorylation in primary podocytes from WT diabetic mice, and overexpression of β-catenin in kidney cortices, which were partially reversed by podocyte Rac1 depletion. Values denote the mean ± s.d.; ^*^P < 0.01 vs. CTL, ^#^*P* < 0.05 vs. WT-STZ. **e** Alterations of Rac1 activities in cultured podocytes. HG led to marked enhancement of Rac1 activity and PAK1 phosphorylation, which were prohibited by Rac1 shRNA transfection. Total Rac1 levels were also reduced by Rac1 shRNA transfection; the lower graph depicts the relative protein levels of p-PAK1. Values denote the mean ± s.d.; ^***^P < 0.01 vs. LG; ^###^*P* < 0.05 vs. HG. **f**, **g** Western blot and real-time PCR illustrated a reduction of both p-cadherin and ZO-1 expression on both protein and mRNA levels after HG stimulation for 48 h. Protein levels of these two were abolished by Rac1 knockdown. PCR showed mRNA levels of p-cadherin and ZO-1 was preserved by Rac1 knockdown or IPA-3 pretreatment; SB203580 also demonstrated a reversal effect on p-cadherin and ZO-1 mRNA expression. Values denote the mean ± s.d.; ^***^P < 0.01 vs. LG; ^###^*P* < 0.05 vs. HG. **h**, **i** Immunofluorescence showed that p-cadherin was intensely stained in the cytoplasm of LG-treated podocytes. HG led to dampened staining of p-cadherin but was reversed by Rac1 knockdown or pretreatment with either IPA-3 or SB203580. ZO-1 was highly expressed at cell–cell contacts under LG conditions. HG resulted to decreased ZO-1 staining in a punctate pattern; this was rectified by Rac1 shRNA transfection or pretreatment with either IPA-3 or SB203580. Cells transfected with Scramble shRNA or pretreated with DMSO were the controls. Scale bar = 200 μm. TG transgenic, WT wild type, CTL control, Rac-sh Rac1 shRNA, Scr-sh scramble shRNA, IPA IPA-3, SB SB203580

In primary podocytes from WT-STZ mice, Rac1 and PAK1 activities were significantly higher compared with normoglycemic controls at 6 weeks (Fig. 3a, b) (*P* < 0.01), which were both abrogated in Rac1-deficient diabetic podocytes (Fig. 3a, b) (*P* < 0.05); p38 was also heavily phosphorylated in WT diabetic podocytes (Fig. 3c) (*P* < 0.01) but suppressed in TG-STZ counterparts at 6 weeks (Fig. 3c) (*P* < 0.05).

**Fig. 4:**
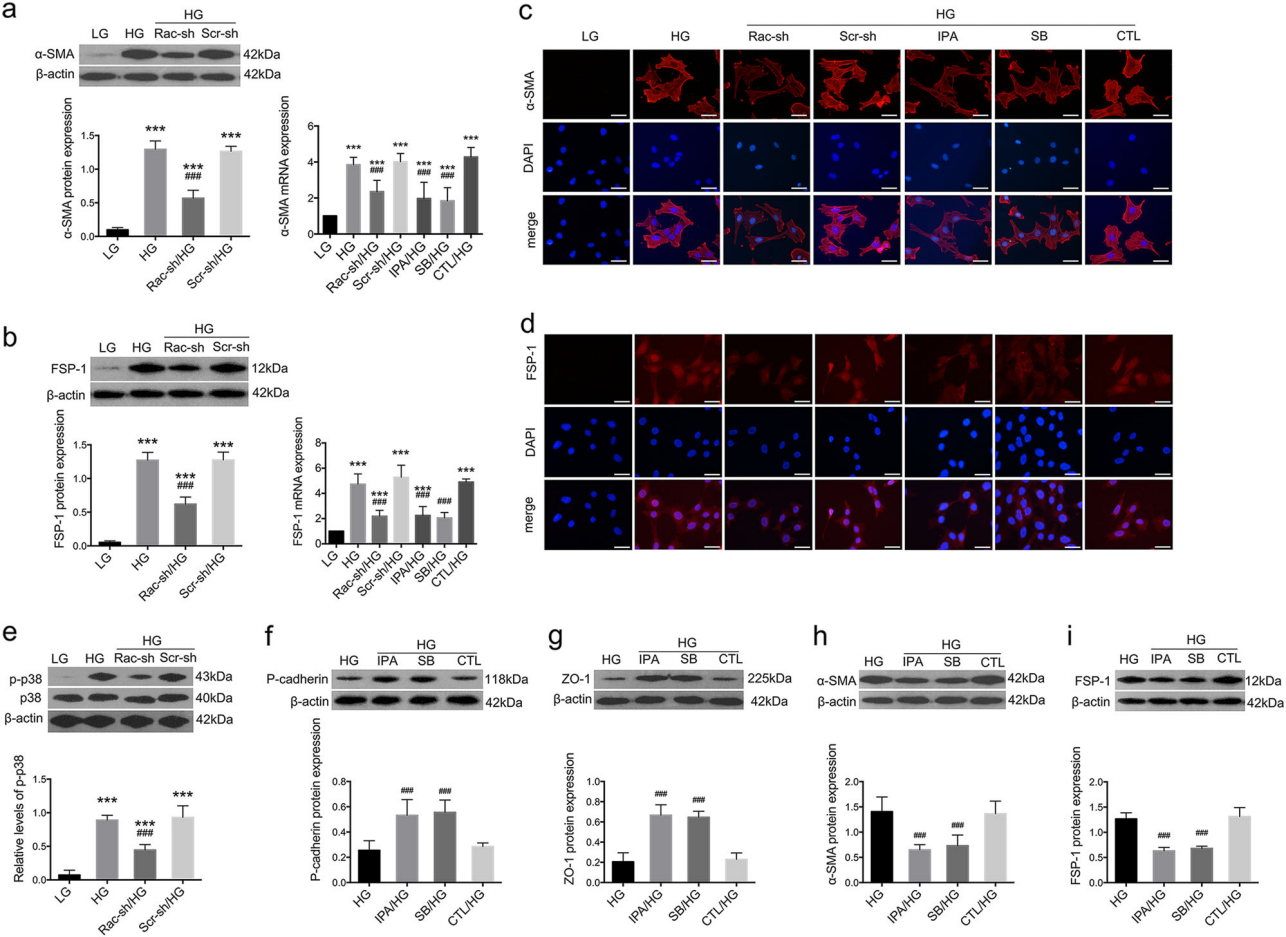
Effects of Rac1/PAK1/p38 pathway on fibroblastic markers under HG conditions. **a**, **b** Both western blot and real-time PCR demonstrated an increase in fibroblastic markers α-SMA and FSP-1 expression on both protein and mRNA levels after HG treatment for 48 h; their protein expression was abolished by Rac1 knockdown; and the mRNA levels of these two markers were abrogated by Rac1 shRNA transfection, or pretreatment of IPA-3 or SB203580. Values denote the mean ± s.d.; ^***^P < 0.01 vs. LG; ^###^*P* < 0.05 vs. HG. **c**, **d** Immunofluorescence showed that α-SMA and FSP-1 were both negatively stained under LG conditions but de novo expressed in the cytoplasm of HG-stimulated podocytes. Decreased staining of both was observed after Rac1 knockdown. Pre-treatment of IPA-1 or SB203580 also demonstrated inhibitory effects on ectopic expression of these two fibroblastic hallmarks, as moderate staining of these two proteins were identified. Cells transfected with Scramble shRNA or pretreated with DMSO were the controls. Scale bar = 200 μm. **e** By western blot, we observed a hyperphosphorylation of p38 after HG stimulation for 48 h, in comparison with counterparts under LG incubation; this was diminished by Rac1 gene knockdown. Values denote the mean ± s.d.; ^***^P < 0.01 vs. LG; ^###^*P* < 0.05 vs. HG. **f**–**i** Effects of inhibitors on protein expression. Pretreatment of IPA-3 or SB203580 led to preserved expression of p-cadherin and ZO-1, but reduced expression of FSP-1 and α-SMA. Values denote the mean ± s.d.; ^***^P < 0.01 vs. LG; ^###^*P* < 0.05 vs. HG. CTL control, Rac-sh Rac1 shRNA, Scr-sh scramble shRNA, IPA IPA-3, SB SB203580

**Fig. 5:**
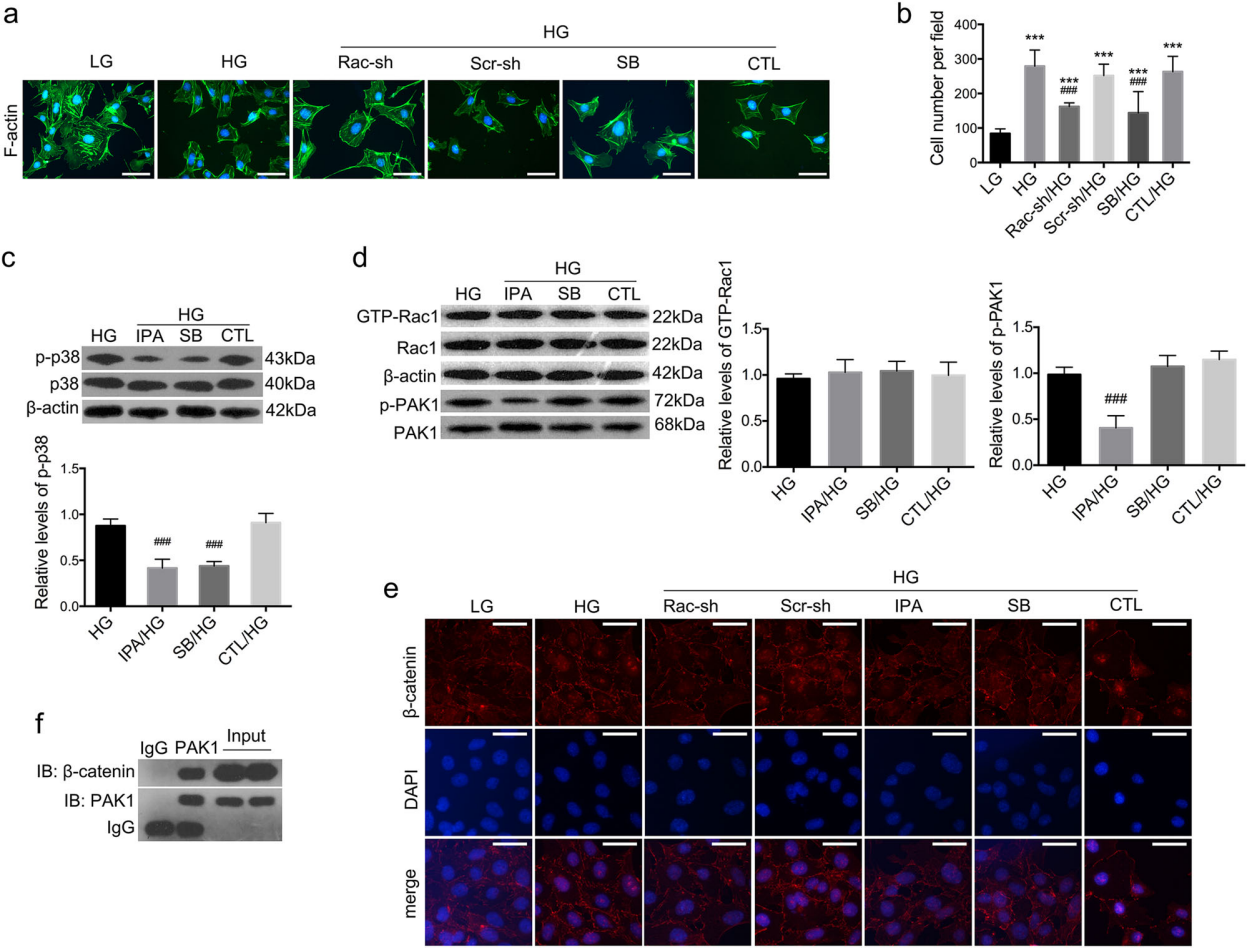
Effects of Rac1/PAK1/p38 cascade on podocyte cytoskeleton rearrangement and β-catenin nuclear accumulation. **a** A rearrangement of F-actin was observed in cultured podocytes, which switched from bundles along the cell axis under LG conditions to densely cortical staining after HG for 48 h. Aberrant distribution was partially reversed by Rac1 shRNA transfection or SB203580 pretreatment. Scale bar = 200 μm. **b** Cell motility by transwell assay. Relatively higher cell motility was driven by HG stimulation, yet abolished by Rac1 shRNA transfection or SB203580 pretreatment. The graph depicted the cell numbers per field passing through the pore. Values denote the mean ± s.d.; ^***^P < 0.01 vs. LG; ^###^*P* < 0.05 vs. HG. **c** Western blot demonstrated phosphorylation levels of p38 in HG-treated podocytes, which was diminished either by IPA-3 or SB203580. Values denote the mean ± s.d.; ^###^*P* < 0.05 vs. HG. **d** GST pull-down showed GTP-Rac1 was not altered after pretreatment of IPA-3 or SB203580. P-PAK1 was affected by the addition of IPA-3, and SB203580 showed limited impacts on PAK activation. Values denote the mean ± s.d.; ^###^*P* < 0.05 vs. HG. **e** Under LG conditions, β-catenin was located in a punctate pattern at cell–cell contacts, which was driven migrating into the nucleus by HG stimulation for 48 h; this was partially abrogated by Rac1 gene knockdown or pretreatment with IPA-3 or SB203580. Scale bar = 200 μm. **f** CO-IP showed the interactions between PAK1 and β-catenin. Immunoblot of initial lysates or immunoprecipitated samples using anti-β-catenin or anti-PAK1 antibodies from podocytes under NC conditions. CTL control, Rac-sh Rac1 shRNA, Scr-sh scramble shRNA, IPA IPA-3, SB SB203580

In vitro, Rac1 and PAK1 were activated in cultured podocytes after HG stimulation for 48 h (Fig. 3e) (*P* < 0.01), despite a relatively higher apoptotic rate compared with cells treated with LG (Figure S), together with remarkable podocyte impairment. Epithelial markers p-cadherin and ZO-1 were reduced (Fig. 3f, g) (*P* < 0.01), whereas injury hallmarks α-SMA and FSP-1 were boosted on both protein and mRNA levels (Fig. 4a, b) (*P* < 0.01). Immunofluorescence illustrated decreased staining of p-cadherin and ZO-1 (Fig. 3h, i), but intensified staining of α-SMA and FSP-1 (Fig. 4c, d) under HG conditions. FITC-phalloidin staining showed HG for 48 h caused F-actin re-organization, from actin bundles along cell axis to densely interwoven filaments at cortical regions on cell periphery (Fig. 5a), together with enhanced cell motility, as was demonstrated by transwell assay (Fig. 5b) (*P* < 0.01). Simultaneously, there was also a remarkable elevation in p38 activation stemming from HG stimulation (Fig. 4e) (*P* < 0.01).

Potential interactions between these proteins were determined. Rac1 knockdown efficiency was first confirmed (Figure S) (*P* < 0.05). Consistent with in vivo data, Rac1 shRNA transfection prevented activation of Rac1, PAK1, and p38, compared with cells without transfection (Figs. 3e and 4e) (*P* < 0.05); and ameliorated podocyte cell markers, evidenced by enhanced expression of p-cadherin and ZO-1 (Fig. 3f, g), and dampened expression of α-SMA and FSP-1 (Fig. 4a, b) (*P* < 0.05). Blunted staining of α-SMA and FSP-1 as well as restored podocyte hallmarks were further confirmed by Immunofluorescence (Figs. 3h, i and 4c, d). Next, we used two chemical inhibitors IPA-3 and SB203580 to target PAK1 and p38 respectively. PAK1 inhibition had reversal effects on both PAK1 and p38 phosphorylation (Fig. 5c, d) (*P* < 0.05), whereas hardly affected Rac1 activity (Fig. 5d) (*P* > 0.05); SB203580 showed limited impacts on Rac1 or PAK1 activation (Fig. 5d) (*P* > 0.05), albeit its inhibitory role in p38 phosphorylation driven by HG (Fig. 5c) (*P* < 0.05). Either IPA-3 or SB203580 were able to preserve expression of p-cadherin and ZO-1, and abrogate expression of dedifferentiation hallmarks desmin, α-SMA and FSP-1 (Fig. 4f–i) (*P* < 0.05). Rac1 shRNA transfection or SB203580 pretreatment reinstated forced F-actin re-assembly, as F-actin bundles at the sub-membrane region were decreased, compared with HG podocytes (Fig. 5a). Concomitantly, transwell assay presented that podocyte hypermotility driven by HG was blunted by Rac1 knockdown or p38 deactivation (Fig. 5b) (*P* < 0.05).

### Rac1/PAK1 signaling stabilized β-catenin by downregulating its ubiquitylation level at Ser675 in podocytes

**Fig. 6:**
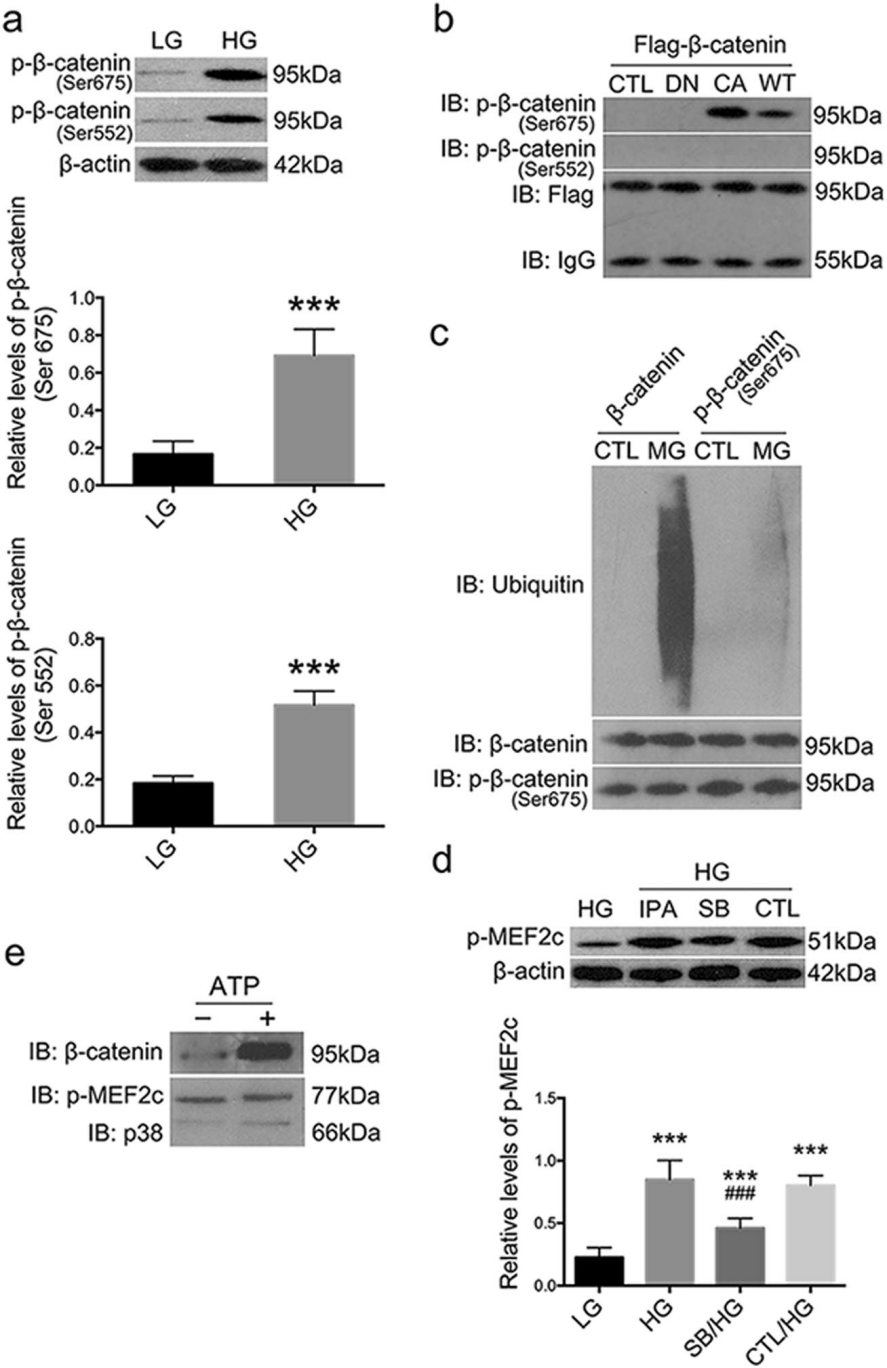
Interplays between Rac1/PAK1/p38 and β-catenin in podocytes under culture. **a** Western blot demonstrated that β-catenin was phosphorylated at Ser675 and Ser552 after HG stimulation for 48 h. Values denote the mean ± s.d.; ^***^P < 0.05 vs. LG. **b** Podocytes were co-transfected with Flag-β-catenin and one of the following plasmids GFP-WT-Rac1, GFP-CA-Rac1, GFP-DN-Rac1, or an empty control vector. Cell lysates were immunoprecipitated with anti-Flag antibodies, and were subjected to immunoblot with the indicated antibodies. **c** HG-stimulated podocytes were treated with MG132 or DMSO for 12 h. Cell lysates were immunoprecipitated with antibodies against total β-catenin or phospho-β-catenin (Ser675), and were subjected to immunoblot with the indicated antibodies. **d** Western blot depicted p-EMF2c was upregulated under HG conditions but was dampened by SB203580. Values denote the mean ± s.d.; ^***^P < 0.01 vs. LG; ^###^*P* < 0.05 vs. HG. **e** The interactions between p38 and β-catenin by in vitro kinase assay. Upper panel was indicative of immunoblot of β-catenin binding to MEF2c following immunoprecipitation; and the lower panel demonstrated the total phosphorylation of MEF2c after incubation with p38 and ATP. CTL control, Rac-sh Rac1 shRNA, Scr-sh scramble shRNA, IPA IPA-3, SB SB203580, DN dominant negative, CA constitutive active, WT wild type

First we found β-catenin expression was elevated in kidney cortices of WT diabetic mice (*P* < 0.01), and reduced in Rac1-deficient hyperglycemic littermates (*P* < 0.01) (Fig. 3d), suggesting that activated Rac1 signaling was an upstream driving force for β-catenin expression in podocytes under diabetic condition; and in HG-cultured podocytes, β-catenin nuclear translocation was provoked by HG stimulation, and was then prohibited by the blockade of Rac1/PAK1/p38 signaling pathway (Fig. 5e). By CO-IP we found PAK1 had physical interactions with β-catenin under normal culture conditions (Fig. 5f), thus we speculated Rac1 might also have physical interplays with β-catenin. Firstly, we showed β-catenin was remarkably phosphorylated at C-terminal Ser552 and Ser675 in HG-podocytes (Fig. 6a) (P < 0.05). Next, by co-transfection of Flag-β-catenin plasmids and GFP-Rac1 mutants, we found WT-Rac1 and CA-Rac1 (V12) were able to induce β-catenin phosphorylation specifically on Ser675, whereas DN-Rac1 (N17) were not (Fig. 6b); in contrast, all three forms of Rac1 had limited phosphorylation effects on Ser552 (Fig. 6b). Moreover, treatment with proteasome inhibitor MG-132 reduced ubiquitylation level of β-catenin when Ser675 residue was markedly phosphorylated under HG conditions, compared with vehicle control (Fig. 6c).

### P38 accounts for HG-induced β-catenin nuclear translocation via activating MEF2c

Also we showed a pronounced activation of MEF2c, a key transcription factor involved in controlling gene expression in several cell types including myocytes, lymphocytes, and neurons^22^, after HG treatment (*P* < 0.01) (Fig. 6d), which was depleted by the addition of p38 inhibitor (*P* < 0.05) (Fig. 6d), when β-catenin nuclear accumulation was also prohibited by SB203580 (Fig. 5e). Next, we performed in vitro kinase assay and showed purified MEF2c, but not β-catenin, could be directly phosphorylated by p38; phosphorylated MEF2c thus strongly bound to β-catenin in vitro (Fig. 6e).

## Discussion

Dysfunction of glomerular podocytes has been implicated as a critical driving force for DN^2, 5^. In the present study, we described a podocyte-specific Rac1-deficient transgenic mouse strain, which exhibited neither embryonic lethality nor spontaneous proteinuria or diabetes. By generating diabetes models in these TG diabetic mice as well as their littermate controls, we were able to show that podocyte-specific Rac1 depletion might be of therapeutic importance to the pathogenesis of DN.

**Fig. 7:**
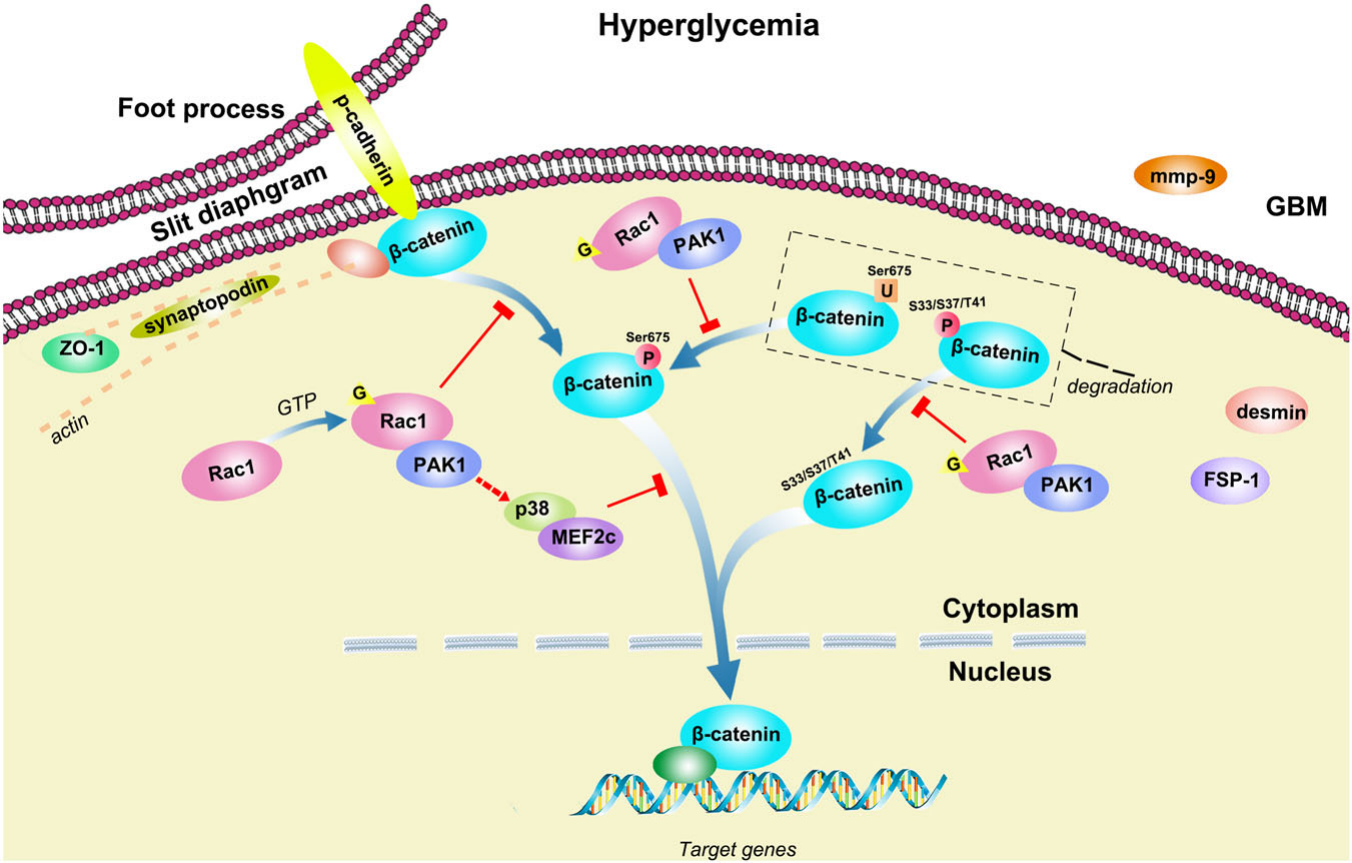
Schematic illustration of Rac1/PAK1/p38 signaling in podocytes in exposure of hyperglycemia. Upon the stimulation of hyperglycemia, Rac1/PAK1 signaling was activated in podocyte; β-catenin was modified, either dephosphorylated at N-terminus or phosphorylated at C-terminus, leading to upregulated level of active β-catenin; the downstream p38 further ensured β-catenin transcriptional activities via activating MEF2c; and active β-catenin contributed to the dysregulation of a series of target genes, and imbalanced expression of SD proteins such as p-cadherin and ZO-1

We showed that consistent hyperglycemia caused striking structural mutations of podocyte-specific proteins p-cadherin and ZO-1, which are also key components of the SD, and a simultaneous retraction of FPs in podocytes at as early as 6 weeks; in diabetic mice where Rac1 was depleted, these alterations were significantly restored. Podocytes elaborate FPs surrounding glomeruli with SDs bridging interdigitations and comprising the final layer of the GFB^23^. SD disruption has been recognized as a key initial theme shared in various kidney diseases arising at the podocyte level^24^. SD generates synchronous signaling for the precise coordination of the neighboring actin-based FPs; damage to the SD was associated with uncoupled signals^24–27^, thus leading to a disrupted cytoskeletal structure, i.e. effaced FPs as was demonstrated here. In vitro, HG induced F-actin rearrangement and cell hypermotility, the latter of which, is recognized as the in vitro manifestation of dynamic reorganization of the FP structure^27^; and these were rectified by Rac1 knockdown. These in vivo and in vitro observations demonstrated Rac1 depletion benefited diabetic podocytes through a major and early attenuation on plasticity of podocyte cytoskeleton via preserving SDs and FPs both structurally and functionally in the challenge of diabetes.

Others and we demonstrated previously that PAK1, a key modulator in regulation of actin polymerization and cell motility in mammalian fibroblasts, served as a major downstream effector of Rac1 in several cell lines including podocytes in vitro^10, 28^. Again, we confirmed PAK1 being a downstream target for Rac1 activation in vitro and in vivo: PAK1 activation was found in podocytes of WT-STZ mice as soon as SD proteins were jeopardized, but was dampened in Rac1 TG-STZ mice. Reportedly, Rac1 contributed to LPS-mediated p38 activation in GDIα knockdown podocytes;^29^ and PAK1 could activate MAPK cascades in oncogenic transformation of a variety of cancer cells^17^. Likewise, we demonstrated p38 being a downstream signaling of PAK1 rather than these two proteins serving as two parallel downstream substrates in injured podocytes upon Rac1 activation, namely a signaling cascade of Rac1/PAK1/p38 in cultured podocytes. Presumably this cascade might as well exist in diabetic podocytes and play a pivotal role based on our in vivo data that simultaneous p38 activation was detected when PAK1 was also activated, and that Rac1 ablation led to reduced activation of both PAK1 and p38 in diabetic glomeruli, further showing reversal of PAK1/p38 activation was an indispensable step to reach the therapeutic effect.

Intriguingly, we found β-catenin expression and nuclear accumulation was under the modulation of Rac1/PAK1/p38 cascade in vitro and in vivo. β-catenin is a key component of Wnt signaling pathway and active β-catenin is found in diverse kidney diseases including focal segmental glomerulosclerosis (FSGS) and DN^19, 30^. Excess cytoplasmic β-catenin is often targeted by a N-terminal phosphorylation/ubiquitylation-mediated degradation system^31–35^. N-terminus dephosphorylation of β-catenin is sufficient to induce stabilized β-catenin migrating into the nucleus^10, 28^. Recent studies indicated β-catenin C-terminal tail constitutes the minimum region necessary for both import and export of cell nucleus in a receptor-free and energy-independent pattern^21^. Consistent with these studies, we demonstrated C-terminal β-catenin was significantly phosphorylated under HG conditions, when β-catenin nuclear accumulation was marked. We further showed that Ser675 at C-terminal β-catenin was a direct phosphorylation target of Rac1 signaling: both WT- and CA-Rac1 (mimicking the pathogenic Rac1 activation upon stimuli) phosphorylated β-catenin at Ser675; this site modification was linked to obliterated ubitquitylation level of β-catenin, which meant more stabilized cytoplasmic β-catenin that could possibly enter the nucleus later on. MEF2c is a key transcription factor involved in controlling gene expression in several cell types including myocytes and neurons^22^. Its expression was elevated in cardiac tissues under experimental diabetic conditions and cardiomyocytes in exposure to high glucose^36^; and in a rodent model of diabetes, increased MEF2c expression was found in the retina and kidney^37^. Likewise, we showed the HG stimuli potentiated MEF2c activation in cultured podocytes. Also we showed that rather than directly bound to β-catenin, p38 interacted with β-catenin in a MEF2c phosphorylation-dependent manner; in other words, MEF2c could only bind to and phosphorylate β-catenin in the premise that p38 was activated. These aforementioned results led us to a potential mechanism that upon the stimuli of hyperglycemia, Rac1/PAK1 signaling was activated in podocyte; β-catenin was then modified, either dephosphorylated at N-terminus or phosphorylated at C-terminus, leading to elevated level of active β-catenin; the downstream p38 further ensured β-catenin transcriptional activities via activating MEF2c; and active β-catenin contributed to the dysregulation of a series of target genes, and imbalanced expression of SD proteins such as p-cadherin, as reported here.

Nuclear β-catenin is responsible for the modulation of a series of EMT genes such as Twist, Snail and Slug^38^. EMT is a phenotypic conversion in epithelial cells, characterized by the loss of epithelial cell markers and the acquisition of fibroblastic hallmarks^4, 10, 27, 37^. We presented in vivo and in vitro the reduction in podocyte epithelial markers (p-cadherin and ZO-1), acquisition of fibroblastic or mesenchymal hallmarks (desmin and FSP-1), and upregulation of β-catenin expression and activation, together with a disordered cytoskeleton (effaced FPs, F-actin reassembly and cell hypermotility), which were, to some extent, prevented by the blockade of Rac1/PAK1/p38 signaling. Mature podocytes, unlike typical epithelial cells, express low levels of an intermediate filament vimentin, and retain a more “motile” cytoskeletal structure applicably assembled and disassembled to meet dynamic requirements of FPs^39–41^. However, we did not show other significant fibroblastic markers such as α-SMA expressed in glomerular podocytes during the course of experimental diabetes in WT or TG mice (not shown). We hypothesized that in vivo diabetic podocytes might undergo a partial EMT or dedifferentiation process; this distinction might be attributed to far more complex circumstances in vivo instead of its direct exposure to the stimuli in vitro. Notably, there was no difference in podocyte numbers between diabetic or nondiabetic mice at this stage of diabetic nephropathy, implying that podocyte were undergoing a relatively early and adaptive mutation rather than apoptosis under such diabetic conditions, which was partially reversible, as indicated in the present study, by Rac1 signaling deficiency. The curative effect was further confirmed by the finding that the onset of proteinuria, which succeeded the podocyte damage, was as well reduced by the rectification of podocyte impairment by the blockade of Rac1 signaling. In addition, we showed Rac1 depletion had a mild attenuation on mesangial areas, implying a pleiotropic effect of podocyte-specific Rac1 deficiency beyond its self-protecting mechanisms. This might be presumably attributed to reduction of mmp-9 in both podocytes and mesangial cells, which played a key role in the extracellular matrix (ECM) turnover^42^.

Albeit the renoprotective effects described above, we showed little impacts of Rac1 inhibition on levels of SBP or BG. Recent studies indicated a signaling cross talk between Rac1 and mineralocorticoid receptor (MR)^8, 43^. Since aldosterone-mineralocorticoid-receptor system has long been recognized as a pivotal player in the salt homeostasis and regulation of blood pressure^44^, it would be of interest to further identify whether there are any compensatory effects after Rac1 depletion. Taken together, we demonstrated podocyte-specific Rac1 depletion attenuated diabetic podocyte injury through the blockade of Rac1/PAK1/p38/β-catenin cascade, which favored DN in following aspects: (a) preserving the structure and function of SDs; (b) rectifying the sophisticated cytoskeleton and FP effacement; (c) reversing podocyte dedifferentiation; (d) reducing early mesangial injury; (e) and reducing proteinuria, without SBP or BG interference (Fig. 7). These findings provided evidence for an early therapeutic potentiality of cell-specific Rac1 deficiency in delaying the development of DN and might also shed some lights on further therapeutic strategies in proteinuric diseases.

## Materials and methods

### Generation of TG mice

The nephrin promoter-Rac1 shRNA was linearized and microinjected into C57BL/6 mouse embryos that were transferred to the oviduct of pseudopregnant recipient mice. By DNA genotyping, 3 of 19 pups were identified positive (Cyagen Biosciences, Guangzhou, China) and were crossed with C57BL/6 mice to generate mice used in the present study. Forward primer: TTCGGTCGACTAGGGATAACAGG; reverse primer: TAATCCAGAGGTTGATTATCGGAA. PCR products (548 bp) were analyzed by agarose gel electrophoresis. The littermates lacking the transgene were control wild type (WT) mice.

### Diabetes models

All animal studies were carried out with the review and approval of the animal care and use committee of Shandong University. At 8 weeks of age, 160 male WT and TG mice randomly allocated into two groups, either intraperitoneally injected with STZ (Sigma-Aldrich, St Louis, MO) dissolved in citrate buffer (50 mg/kg for 5 consecutive days) (pH 4.5) or citrate buffer alone. Hyperglycemic state was monitored and maintained on a standard rodent diet with water ad libitum. SBP was examined by tail-cuff manometry (BP-2000, Visitech Systems, NC). Urine was collected for determination of urinary protein when mice were weighed and placed in metabolic cages for 24 h. At the end of 4, 6, 8, and 12 weeks, mice were sacrificed and kidneys were harvested for following experiments.

### Transmission electron microscopy

Small pieces of renal cortex were fixed in glutaraldehyde and osmic acid, dehydrated with an ethanol gradient, soaked in ethoxyline resin overnight, and mounted at 60 °C for 48 h. Ultrathin sections were cut and viewed under H-7500 TEM (Hitachi, Tokyo, Japan).

### Cell culture

Isolation of glomeruli was done by three-step sieving (250, 100, and 70 μm) of kidney cortices^45^. Isolated glomeruli were maintained on type I collagen in RPMI 1640 (10% FBS, 100 U/ml penicillin and 100 mg/ml streptomycin) (Life Technologies, CA, USA). After 7 days, glomeruli were removed and cells were trypsinized and passed through a 40-μm-pore size sieve to remove mesangial and endothelial cells^46^. Podocytes identity was then done by synaptopodin and WT-1 immunofluorescence. Conditionally immortalized mouse podocytes were kindly provided by Professor Peter Mundel (Massachusetts General Hospital, Boston, MA, USA) via Professor Jie Ding (Peking University). Podocytes were cultured as described previously^10, 46^.

### Transfection and RNA interference

Rac1 shRNA 5′−GACGTGTTCTTAATTTGCTT-3′, a scrambled shRNA 5′−GCGCGCTTTGTAGGATTCG-3′, Flag-β-catenin vectors, GFP-WT-Rac1, GFP-constitutive active (CA)-Rac1 (CA) (V12), GFP-dominant negative (DN)-Rac1 (N17), and an empty vector, were from Cyagen Biosciences (Guangzhou, China). Transfection was performed (1 × 10^6^ cells/well) with indicated plasmids using Lipofectamine3000 reagent (Invitrogen, Life Technologies Corporation). After Rac1 shRNA incubation, podocytes were either treated with low glucose (LG) (5.6 mM) or HG (30 mM) for an additional 48 h. Cells transfected with scrambled shRNA and incubated with HG for 48 h were controls.

### Measurement of Rac1 activity

Rac1 activation was assessed using a pull-down assay kit according to the manufacturer’s instructions (#80501, NewEast Biosciences, PA, USA). Briefly, cells were harvested and then lysed with assay/lysis buffer containing protease inhibitors on ice. The cell lysates were then centrifuged and protein content was estimated using the Bio-Rad protein assay. To analyze active Rac1 levels, each sample was adjust the volume of to 1 ml with 1× assay/lysis Buffer Lysate and incubated with 1 μl anti-active Rac1 monoclonal antibody, bound with protein A/G agarose beads for 1 h at 4 °C. The GTP-bound forms of Rac1 in the pull-down samples were precipitated, run in a 10% SDS-PAGE gel and subjected to western blot.

### Co-immunoprecipitation

Co-IP was performed using Universal Magnetic Co-IP Kit according to the manufacturer’s instructions (#54002, Active Motif).

### In vitro kinase assay

Recombinant GST-p38 and GST-myocyte-specific enhancer factor 2C (MEF2c) (Abnova, Heidelberg, Germany) were incubated in kinase buffer for 3 h at 30 °C with or without ATP addition (NEB, Ipswich, USA), followed by 6× His-β-catenin (Cyagen Biosciences, Guangzhou, China), glutathione-agarose beads (Thermo, IL, USA), and NP-40 lysis buffer (Solarbio, Beijing, China). Beads were heated, run in SDS-PAGE gel, and subjected to western blot.

### Western blot

The protocol was described previously^10^. Primary antibodies used were as follows: p-cadherin (Santa Cruz, CA, USA), FSP-1, α-SMA, β-catenin, mmp-9, Rac1 (Abcam, MA, USA); phospho-β-catenin (Ser552), phospho-β-catenin (Ser675), phospho-p38 (Thr180/Tyr182), p38, PAK1 and phospho-PAK1 (Thr423) (Cell Signaling Technology, MA, USA), β-actin (Proteintech, IL, USA), ZO-1 (Thermo Fisher Scientific, IL, USA) and desmin (Immunoway). Bands were detected using ECL system (Amersham Biosciences, Piscataway, NJ, USA) and Bio-Rad electrophoresis image analyzer (Bio-Rad, Hercules, CA, USA).

### Real-time reverse transcriptase PCR

Total RNA extraction and reverse transcription, and amplification were described as previously^10^. Primers were from Sangon Biotech (Shanghai, China). Sequences were designed as follows (Table 2).

**Table 2:** Primers for real-time PCR

Gene	Primer	Sequence
P-cadherin	Sense	5′-GTTTGAGCCGCAGAAGTATGA-3′
	Antisense	5′-GAGTGGTGATGGTGAAATGGT-3′
ZO-1	Sense	5′-GAGCTACGCTTGCCACACTGT-3′
	Antisense	5′-TCGGATCTCCAGGAAGACACTT-3′
α-SMA	Sense	5′-TGTGTGAAGAGGAAGACAGCA-3′
	Antisense	5′-TCCAACCATTACTCCCTGATG-3′
FSP-1	Sense	5′-TGTGTCCACCTTCCACAAATAC-3′
	Antisense	5′-ACTTCATTGTCCC TGTTGCTGT-3′
β-actin	Sense	5′-AAGACGAGGAGGAACTGAAC-3′
	Antisense	5′-CAAATCGGA CAACAAGACG-3′
Rac1	Sense	5′-GTAAAACCTGCCTGCTCATCA-3′
	Antisense	5′-GGGACGCAATCTGTCATAATC-3′

### Histology, immunohistochemistry, and immunofluorescence

Kidney sections were stained via PAS method. Podocyte counts were determined by WT-1 staining. For immunofluorescence, kidney sections or fixed cells were immunostained with primary antibodies at 4 °C overnight and Dylight 594- or 488-conjugated IgG the next day; FITC-phalloidin (50 μg/ml) was used for F-actin staining. All analyses were performed in a blinded fashion. Images were captured on an inverted phase/fluorescence microscope (Leica Microsystems GmbH, Wetzlar, Germany).

### Transwell migration assay

Transwell cell-culture inserts (8 μm) (Corning, MA, USA) were placed in RPMI1640 (10% FBS) in lower compartment. Podocytes were seeded in upper chambers (1 × 10^4^/ml), which were allowed to attach at 37 °C for 48 h. Non-migratory cells were removed; migrated cells were stained with haematoxylin. Cell number in the center of a membrane (one field) was counted using phase contrast microscopy (Leica Microsystems GmbH). Data presented denote the mean ± s.d. of three independent experiments.

### Statistics

Experiments were performed at least three times. Values were reported as mean ± s.d. Data were analyzed using SPSS 19.0 software. Statistical significance was assessed using Student’s *t*-test, and one-way ANOVA and LSD-*t* test; and *P* < 0.05 were considered to be statistically significant.

## Electronic supplementary material

Supplementary figure

Figure S
