# Supplementary figures and images for: Podocyte-specific Rac1 deficiency ameliorates podocyte damage and proteinuria in STZ-induced diabetic nephropathy in mice
Source: Cell Death Dis. 2018 Mar 1;9(3):342. doi: 10.1038/s41419-018-0353-z (PMC5832796; doi:10.1038/s41419-018-0353-z)

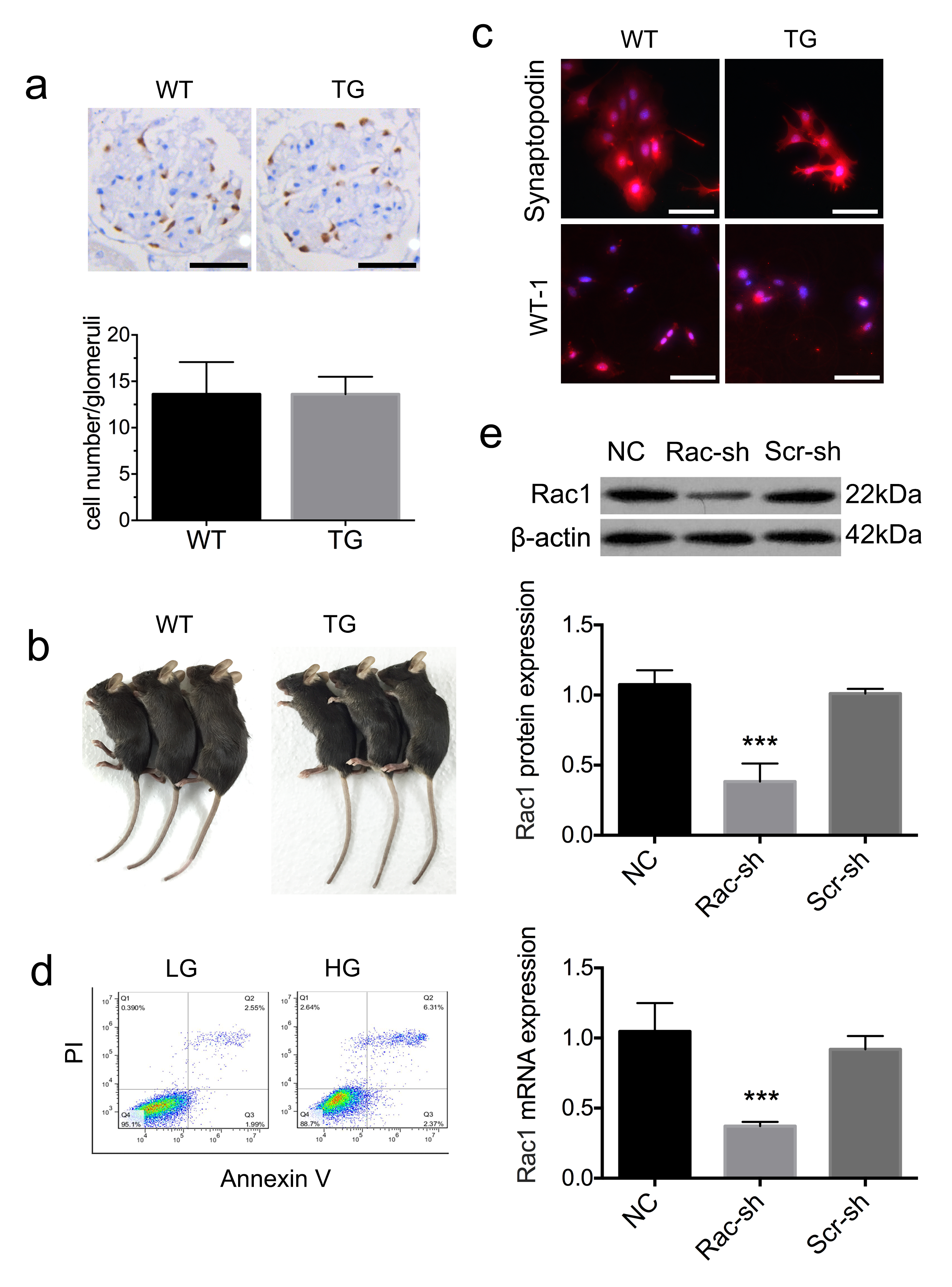

Supplement: Supplementary file 1 — Supplementary figure [file 41419_2018_353_MOESM1_ESM.tif]
