# Supplementary material for: Podocyte-specific Rac1 deficiency ameliorates podocyte damage and proteinuria in STZ-induced diabetic nephropathy in mice
Source: Cell Death Dis. 2018 Mar 1;9(3):342. doi: 10.1038/s41419-018-0353-z (PMC5832796; doi:10.1038/s41419-018-0353-z)
Supplement: Supplementary file 2 — Figure S [file 41419_2018_353_MOESM2_ESM.docx]

**Figure S (a**) Podocyte numbers were measured by WT-1 staining. No significant differences between WT and TG mice in podocyte counts were demonstrated. Quantification of WT-1-positive cells per glomerular area was presented in the graph. Values denote the mean ± s.d.; *P*>0.05 vs. WT. Scale bar=100μm. (**b**) No distinguishable differences were observed in appearance between TG and WT mice. (c) Podocytes identity was done by synaptopodin and WT-1 immunofluorescence. Scale bar=200μm. (**c**) Apoptotic rates by flow cytometry. HG treatment for 48h resulted to a relatively higher apoptotic rate than that of cells under LG conditions. (**d**) Both western blot and real-time PCR demonstrated a relatively high knockdown efficiency of Rac1 gene after shRNA transfection for 24h. Values denote the mean ± s.d.; ^***^*P*<0.05 vs. NC. TG, transgenic; WT, wild type; CTL, control.
